# Methicillin-resistant *Staphylococcus aureus* nasal colonization in people living with HIV and healthy people in Kathmandu, Nepal

**DOI:** 10.2144/fsoa-2021-0103

**Published:** 2021-11-26

**Authors:** Samjhana Kapali, Anil Pokhrel, Anup Bastola, Reshma Tuladhar, Dev Raj Joshi

**Affiliations:** 1 Central Department of Microbiology, Institute of Science & Technology, Tribhuvan University, Kathmandu, 44613, Nepal; 2 Department of Dermatology & Sexually Transmitted Infections, Sukraraj Tropical & Infectious Disease Hospital, Kathmandu, 44600, Nepal

**Keywords:** *mecA*, MRSA, multi-drug resistance, PLHIV, *PVL*, *Staphylococcus aureus*

## Abstract

**Aim:**

This study aimed to compare methicillin-resistant *Staphylococcus aureus* (MRSA) nasal colonization in people living with HIV (PLHIV) and healthy people from Kathmandu.

**Methods:**

MRSA isolated from 400 nasal swabs was screened using a cefoxitin disc and confirmed by the presence of the *mecA* gene.

**Results:**

MRSA nasal carriers among the PLHIV and control cohorts were 3.5% (7 out of 200) and 5.0% (10 out of 200), respectively. All the MRSA from PLHIV and most of MRSA from healthy controls were *PVL* positive. Longer duration of antiretroviral therapy significantly reduces the risk of MRSA nasal colonization in PLHIV.

**Conclusion:**

There is no significant difference in MRSA nasal colonization in PLHIV and healthy controls in this study region.

## Lay Abstract

People living with HIV (PLHIV) are more prone to the colonization of *Staphylococcus aureus* (*S. aureus*) and therefore to methicillin resistance *S. aureus* (MRSA). In this study, we compared the MRSA colonization in PLHIV with that in healthy people in the Kathmandu Valley and found no significant variation in the prevalence of MRSA in the two study groups. We found that longer antiretroviral therapy reduces the risk of MRSA colonization in PLHIV. Recent history of hospitalization increases the chances of MRSA colonization among PLHIV as well as healthy people.

*Staphylococcus aureus*, established as a common colonizer of the anterior nares, can cause minor skin infections to life-threatening diseases [1,2]. Nasal colonization of *S. aureus* appears to be detrimental in persistent carriers [3] and immunocompromised individuals, such as people living with HIV/AIDS [4], patients undergoing dialysis [4] and surgery [5]. It is asserted that around 20.0% of the general population are persistent carriers of *S. aureus* and 30.0% are intermittent carriers [6–8]. Individuals colonized by *S. aureus* and methicillin-resistant *S. aureus* (MRSA) are likely to develop an infection [9,10]; furthermore, individuals colonized with MRSA are at higher risk than those with *S. aureus* [11].

Since the first report in the 1960s, MRSA has been widespread in the hospital setting [12] and community [13–15], leading to augmentation of complications in the treatment of *S*. *aureus* infections [16]. The *mecA* gene, which confers resistance to methicillin, is harbored by both hospital-associated (HA-MRSA) and community-associated [17] MRSA (CA-MRSA). CA-MRSA is mainly associated with skin and soft tissue infections (SSTI), in the absence of any kind of healthcare exposure [18]. SSTI is the most common type of MRSA infection in HIV-infected people and PVL toxin is related to necrotizing infection [19].With the emergence of antimicrobial resistance and acquisition of virulent genes by bacteria, treatment due to opportunistic pathogens owing to the weak immune system of the immunocompromised host such as people living with the human immunodeficiency virus (PLHIV) has become intricate [20]. Human immunodeficiency virus (HIV) reduces immunity by lowering the number and functional efficacy of CD4 helper lymphocytes [20], leading to a greater risk of opportunistic infections and other infections [21].

PLHIV are six to eighteen times more prone to MRSA infections than the general population [22,23]. The MRSA prevalence among PLHIV ranges from 2.3 to 69.1%, especially in South and East Asia and the western-Pacific regions [24]. MRSA carriage may lead to subsequent infection with an increased rate of morbidity and mortality [25]. As far as we know, despite the government of Nepal's efforts to reduce the burden of HIV/AIDS by collaborating with many national and international organizations, the most recent and most reliable statistics on PLHIV in Nepal are not accessible. It is estimated that there are more than 50,000 cases of PLHIV in Nepal [26] but unfortunately, less than 50% are enrolled in treatment [27]. Substantial information on *S. aureus* colonization in PLHIV is still not available in Nepal. We hypothesized that there is a higher frequency of nasal colonization of MRSA in PLHIV compared with people. Therefore, this study was intended to compare the prevalence of MRSA in PLHIV and healthy people as a control group.

## Methods

### Study site, design & sampling

A prospective cross-sectional study was conducted from July to December 2019 on PLHIV attending the antiretroviral therapy (ART) center of Sukraraj Tropical and Infectious Disease Hospital (Kathmandu, Nepal) and healthy people residing in the Kathmandu Valley. A total of 400 subjects were enrolled, with 200 from each study group. The nasal swab from both anterior nares of a patient was collected by using sterile cotton swabs pre-moistened with sterile normal saline, labeled and transported to the laboratory in Stuart transport media within 2–3 h [28].

PLHIV with a history of HIV infection (seropositive) as per their clinical records at the ART center were included in the study. Seemingly healthy people, who were enrolled in their respective households, were interviewed for any existing health issues and the present use of antibiotics prior to enrolling in the study. People with health complications or other immunodeficiency conditions such as renal transplant, cancer, diabetes, liver cirrhosis, malignancy and subjects below 19 years of age from both the study groups were excluded from the study. People who were receiving antibiotic treatment for any infection during the time of data collection were also excluded.

We used n = z^2^pq/d^2^ to calculate the minimum sample size. Because there had been no population based study or pilot survey carried out before, a previously published similar cross-sectional study [29] was used as reference to calculate the sample size for PLHIV and found it to be 183, which was increased to 200. We did not employ such a formula to calculate the minimum sample size for people, but we chose 200 to make comparisons with 200 PLHIV individuals easier.

### Sample processing & identification of *S. aureus*

The nasal swabs were inoculated into blood agar (BA) and mannitol salt agar (MSA) and incubated aerobically at 37°C for 24 h. Colonies morphologically suggestive of *S. aureus* were confirmed by Gram staining, catalase test, coagulase test and DNase test.

### Antimicrobial susceptibility test

A single colony of *S. aureus* isolates from each plate was subjected to antimicrobial susceptibility test by the Kirby Bauer disc diffusion method with the antibiotics: amoxicillin (10 μg), cefoxitin (30 μg), ciprofloxacin (5 μg), clindamycin (2 μg), cotrimoxazole (25 μg), erythromycin (15 μg), gentamicin (10 μg), penicillin (10 μg) and tetracycline (30 μg). Methicillin resistance was detected using a 30 μg cefoxitin disc. An isolate with a diameter of the zone of inhibition of ≤21 mm with cefoxitin was considered MRSA [30]. *S. aureus* isolates resistant to at least one antibiotic in three or more antimicrobial categories were classified as multidrug resistant (MDR) [31].

### Amplification & detection of *mecA* & *PVL* genes

Genomic DNA was extracted from an overnight culture of *S. aureus* in Luria-Bertani (LB) broth by the phenol: chloroform: isoamyl alcohol extraction method [32]. The *mecA* and the *PVL* genes were amplified using the primer described previously [33,34]. A 25 μl reaction mixture consisting of 13 μl master-mix (Thermo Fisher Scientific, India), 8 μl nuclease-free water, 0.5 μl each forward and reverse primers and 3 μl template DNA was subjected to the amplification conditions.

The PCR products were detected by agarose gel electrophoresis (1.2% agarose gel for *PVL* gene detection and 2.5% for *mecA*, gel with ethidium bromide (0.5 μg/ml) incorporated) at 100 V [33,34] and visualized under UV transilluminator.

### Data analysis

The data on participants' demographics obtained through structured questionnaires and the bacterial isolates identified by laboratory techniques were entered in and analyzed by a statistical analysis tool (SPSS 21.0 Version, IBM, NY, USA). The statistical significance of the relationship among different variables was calculated by chi-square test, one-way ANOVA and binary logistic regression analysis as appropriate. The tests with a p-value < 0.05 were considered statistically significant (at 95% CI).

## Results

### Demographic & clinical features of the study groups

The average ages of the PLHIV and the healthy controls included in the study were 41.34 ± 10.212 years and 36.16 ± 14.608 years, respectively. An equal proportion of males and females were observed (Table 1). Twenty-three percent of PLHIV were under ART for <6 months and 35.0% for more than 5 years. The CD4 count of <200 cells/μl was observed in 9.0% of PLHIV. Sixteen percent of PLHIV had a history of hospitalization within the past 6 months (Table 1).

**Table 1.** Demographic and clinical features of study groups.

Variables		
	PLHIV (n = 200)	Healthy controls (n = 200)
Age (years), mean ± SD	41.34 ± 10.212	36.16 ± 14.608
**Gender**		
Male	107 (53.5%)	99 (49.5%)
Female	93 (46.5%)	101 (50.5%)
**Residence**		
Urban municipality	132 (66.0%)	185 (92.5%)
Rural municipality	68 (34.0%)	15 (7.5%)
**Marital status**		
Married	153 (76.5%)	111 (55.5%)
Unmarried	15 (7.5%)	86 (43.0%)
Widow	25 (12.5%)	3 (1.5%)
Divorced	7 (3.5%)	0
**ART duration**		
<6 months	46 (23.0%)	-
6 months–5 years	84 (42.0%)	-
>5 years	70 (35.0%)	-
**CD4 cell count (cells/μl)**		
<200	18 (9.0%)	-
≥200	182 (91.0%)	-
**Hospitalization in past 6 months**		
Yes	32 (16.0%)	8 (4.0%)
No	168 (84.0%)	192 (96.0%)

ART: Antiretroviral therapy; PLHIV: People living with HIV.

### Antibiotic susceptibility pattern of the isolates

**Figure 1.**
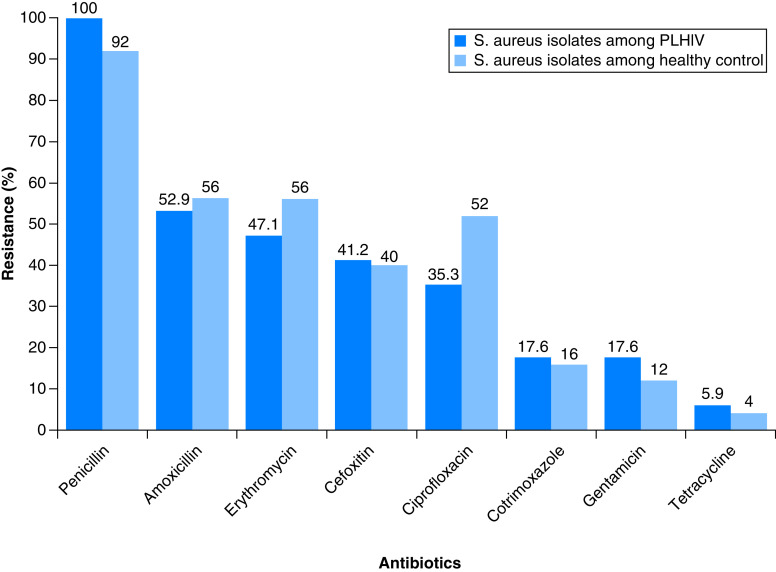
Percentage of *Staphylococcus aureus* resistant to tested antibiotics, from people living with HIV and healthy controls. PLHIV: People living with HIV.

From 400 nasal swabs, *S. aureus* growth was detected in 42 out of which 17 were from PLHIV and 25 were from the healthy control. The antimicrobial susceptibility test pattern of *S. aureus* isolates is depicted in Figure 1. Out of a total of 42 *S. aureus* nasal carriers, 17 were MRSA based on a cefoxitin disc test and 25 were MSSA carriers (Table 2). Seven MRSA and 10 MSSA were from PLHIV. No significant difference in the prevalence of MRSA between the study cohorts was observed (p-value: 0.457, Table 2). Almost equal proportions of *S. aureus* (19 isolates from males) and MRSA (7 isolates from males) were isolated from males and females.

**Table 2.** Methicillin-resistant *Staphylococcus aureus* and multidrug resistant *Staphylococcus aureus* among study participants.

*S. aureus* (n = 42)	Sample type		Total	χ^2^ p-value
	PLHIV (n)	Healthy controls (n)		
MRSA	7	10	17	0.457
MDR	11	16	27	0.319

MDR: Multidrug resistant; MRSA: Methicillin-resistant *Staphylococcus aureus*; PLHIV: People living with HIV.

All *S. aureus* isolates from PLHIV and most of the isolates from the control group were resistant to penicillin, while the least resistance was observed for tetracycline in both groups. Similarly, MDR *S. aureus* accounted for 27 of the total isolates and seventeen of these MDR were MRSA (Table 2). The remaining ten MDR strains were methicillin-susceptible *S. aureus* (MSSA), of which four were from PLHIV and six were from the healthy control. Eleven isolates out of 17 isolates from PLHIV were MDR, and 16 isolates out of 25 isolated from the control group were MDR. A statistically significant difference was not observed in the prevalence of MDR *S. aureus* between the two groups (p-value: 0.319, Table 2).

The AST pattern of MRSA is shown in Table 3. There was no significant difference in the resistance pattern of MRSA between the two study groups (Table 3). Out of the total *S. aureus*, 9 showed inducible macrolide-lincosamide-streptogramin B (iMLSB) including 3 from PLHIV and 6 from healthy controls as revealed by D-test phenotyping.

**Table 3.** Antibiotic resistance of methicillin-resistant *Staphylococcus aureus* in people living with HIV and healthy controls.

Antibiotics (μg)	PLHIV (n = 7)	Healthy controls (n = 10)	p-value (One-way ANOVA)
	Resistant (n)	Resistant (n)	
Amoxicillin (10)	7	10	0.244
Clindamycin (2)	0	1	0.420
Erythromycin (15)	5	5	0.146
Penicillin (5)	7	10	0.244
Gentamicin (10)	2	1	0.354
Cotrimoxazole (25)	2	1	0.354
Tetracycline (30)	0	0	-
Ciprofloxacin (5)	3	8	0.130

### Detection of *mecA* & *PVL* genes

All the phenotypic MRSA isolates from both the study groups harbored the *mecA* gene (Table 4), which was absent in MSSA isolates from both groups. Among 42 isolates, the *PVL* gene was detected in 21 isolates, of which six isolates were MSSA (Table 4). There was no statistically significant difference in the presence of *PVL*-positive MRSA between the two groups (*χ*^2^ test p-value: 0.823).

**Table 4.** Distribution of *mecA* and *PVL* genes among methicillin-resistant *Staphylococcus aureus* isolates.

Sample type	Phenotypic-MRSA isolates (n)	*mecA*-positive MRSA (n)	*PVL*-positive MRSA (n)	*PVL*-positive MSSA (n)
PLHIV	7	7	7	5
Controls	10	10	8	1
Total	17	17	15	6

MRSA: methicillin-resistant Staphylococcus aureus; PLHIV: People living with HIV; MSSA: Methicillin-susceptible *Staphylococcus aureus*.

### Risk factors associated with MRSA nasal colonization in the study groups

In PLHIV with a CD4 count of <200 cells/μl, MRSA colonization (22.2 in PLHIV and 5.0% in healthy control) and *S. aureus* colonization (27.8 in PLHIV and 12.5% in healthy control) were higher than in healthy controls (Table 5).

**Table 5.** Comparison of methicillin-resistant *Staphylococcus aureus* colonization in the study cohort.

Sample type	CD4 count (cells/μl)	Frequency	*S. aureus* nasal colonization (n)	MRSA nasal colonization (n)
PLHIV	<200	18	5	4
	≥200	182	12	3
Healthy controls	-	200	25	10

MRSA: methicillin-resistant Staphylococcus aureus; PLHIV: People living with HIV.

The nasal colonization rate of MRSA was higher in PLHIV with a CD4 count of <200 cells/μl but was not statistically significant (p = 0.823) (Table 6). The MRSA nasal colonization was found to be the highest (10.9%) in the participants with ART duration of less than 6 months, but not reported in the participants receiving ART for more than 5 years. The longer ART duration lowers the odds of nasal colonization by MRSA (p = 0.014) (Table 6). The MRSA nasal colonization rate was statistically significant in both cases of PLHIV (p = 0.002) and healthy controls (p = 0.023) with a history of hospitalization within the past 6 months (Table 6).

**Table 6.** Associated risk factors for methicillin-resistant *Staphylococcus aureus* colonization.

Risk factors	PLHIV		Healthy controls	
	OR (95% CI)	p-value	OR (95% CI)	p-value
ART duration	0.156 (0.035–0.688)	0.014	-	-
History of hospitalization in past 6 months (No/Yes)	15.370 (2.838–83.258)	0.002	7.667 (1.332–44.113)	0.023
CD4 cell count (cells/μl)				
<200	0.997 (0.971–1.024)	0.823	-	-
≥200	0.996 (0.990–1.002)	0.225^1^	-	-

ART: Antiretroviral therapy; OR: Odds ratio; PLHIV: People living with HIV.

## Discussion

This study recorded a lower prevalence of *S. aureus* in PLHIV and healthy controls as compared with the prevalence reported by Neupane *et al.* [29] from the same region of Nepal. However, they enrolled HIV-negative subjects who attended the hospital as their control group, in contrast to community dwellers as the control group in this study. On the contrary of our finding that there was no significant difference in *S. aureus* nasal colonization between PLHIV and healthy individuals, a study from India [35] reported a significantly higher rate of *S. aureus* nasal colonization among HIV-infected individuals compared with HIV-uninfected individuals. This study reported a relatively higher prevalence of MRSA among PLHIV and healthy controls, even though the difference between the two study groups was not statistically significant (p-value: 0.457), previous studies elsewhere have also reported the absence of statistical significance of MRSA colonization between the cohorts of PLHIV and HIV negative [36,37]. Some studies from the USA, however, reported statistically significant higher isolation of MRSA among HIV-seropositive individuals compared with the HIV-seronegative control group [38,39]. The lower isolation rate of *S. aureus* among PLHIV might be due to the indirect effect of ART because ART helps to increase CD4 cell count [23]. The decreasing prevalence of MRSA as recorded in this study indicates improved hygiene practice and hygiene disposition of individuals could be a factor determining the rate of nasal carriage of MRSA among PLHIV [40]. The difference in rates of isolation of MRSA in different studies might be due to the variations in sample collection sites and survey time [41], implementation of the infection control program and use of antibiotics according to hospitals and countries [42]. The isolation of *S. aureus* is affected by the site of sample collection, higher rates of *S. aureus* isolation have been reported from nasal specimens compared with other sites [43,44], this may be because anterior nares are the common ecological niche for *S. aureus*, besides, the use of pre-enriched media for isolation could influence the isolation rate of *S. aureus* as observed in other specimens [45]. Hidron *et al.* [39] hypothesized that the use of trimethoprim-sulfamethoxazole (also called cotrimoxazole) for prophylaxis of *Pneumocystis carinii* pneumonia possibly reduces the colonization of MRSA in PLHIV, and this statement is supported by other researchers as well [13,46]. In Nepal, cotrimoxazole prophylaxis is given to HIV-infected adults with a CD4 count of <500 cells/μl, adults who have had an episode of *Pneumocystis carinii* pneumonia and individuals with symptomatic HIV infection [47].Comorbidities in PLHIV attending the ART center and people were not assessed in this study, and all the subjects were screened only once for *S. aureus* or MRSA colonization. MRSA isolates could not be genotypically characterized owing to budget and resource constraints. The results of this study may not be typical of the entire population of the Kathmandu Valley because it is a cross-sectional study with a smaller sample size.

We recorded a higher prevalence of MRSA among PLHIV with a CD4 cell count of <200 cells/μl as compared with those with a CD4 cell count of ≥200 cells/μl. This study also showed that CD4 cell count does not significantly influence the rate of MRSA detection in the nasal carriage of *S. aureus* from PLHIV, which is also supported by Shet *et al.* [9] who suggested that MRSA colonization in PLHIV might not be dependent on CD4 T-lymphocytes. In contrast, some other researchers have documented that the lower CD4 count is associated with MRSA colonization in PLHIV [48,49]. However, MRSA colonization in PLHIV with a CD4 count of <200 cells/μl was fourfold higher compared with the healthy controls, and this infers that high CD4 T-cell counts have a protective effect and PLHIV with a low CD4 count are more vulnerable to developing colonization due to MRSA. Similar results were documented from Singapore [49], Ethiopia [28] and Nepal [29].

Corresponding with the fact that HIV infected people who received ART in the past years had a significantly decreased risk of MRSA colonization or infection [23,50,51], this study also establishes that longer ART duration lowers the odds of MRSA nasal colonization (p-value: 0.014; OR: 0.156; 95% CI: 0.035–0.688). The ART vividly increases CD4 cell count while simultaneously decreasing the viral load among people under ART, thus reduction of the *S. aureus* nasal colonization could be an indirect effect of ART [23]. Nevertheless, the association between MRSA colonization in PLHIV and the ART-treatment regime is still not well established [38,39,46]. As supported by previous studies [22,23,52–54], we documented that prior hospitalization in the past 6 months in both PLHIV (p-value 0.002; OR: 15.370; 95% CI: 2.838–83.258) and the control group (p-value: 0.023; OR: 7.667; 95% CI: 1.332–44.113) significantly increased the likelihood of MRSA nasal colonization, suggesting the recent history of hospitalization as a predictor for MRSA colonization. This can be attributed to the fact that hospitalization might facilitate MRSA colonization via direct contact with infected individuals or contaminated objects. However, some researchers found that the history of hospitalization is not significantly associated with colonization in both the study cohorts [35].

The high prevalence of MDR *S. aureus* in this study is a matter of concern. The practice of self-medication, incomplete antibiotic courses and abuse of antibiotics are not uncommon in Nepal [55], these reasons, along with antibiotic misuse in animal husbandry in Nepal, may have contributed to acquiring MDR strains [56]. The highest percentage of *S. aureus* and MRSA were resistant to penicillin and the least resistance of *S. aureus* and MRSA to tetracycline and cotrimoxazole was observed in the case of both the study groups in the present study and similar results have been reported elsewhere [17,36,43,57]. The studies also suggested that the observed low rates of resistance to cotrimoxazole might suggest that the colonization is caused by CA-MRSA rather than HA-MRSA [17,43,58]. However, cotrimoxazole resistance was higher among PLHIV than among people, which could be the result of cotrimoxazole prophylaxis provided to PLHIV for treatment of opportunistic infections [59], and cotrimoxazole prophylaxis is given to HIV-infected adults with a CD4 count of <500 cells/μl in Nepal [47]. The lower ciprofloxacin resistance among the MRSAs from the PLHIV might be due to the smaller sample size in this study. This relatively high ciprofloxacin sensitivity in HIV positive subjects have been reported by a few other studies [60,61], furthermore, this lower resistance to ciprofloxacin among MRSA may not be exclusively related to PLHIV. High ciprofloxacin sensitivity has been reported among CA-MRSA by Fridkin *et al.* in the USA [62].In concordance with a previously published report elsewhere [63], this study indicates that the iMLSB strain is common in the Nepalese setting. High inducible clindamycin resistance was observed among healthy controls than in PLHIV in this study, possibly because of the increasingly prescribed clindamycin by health professionals in outdoor clinics [64]. Besides, underlying comorbidities such as diabetes mellitus, renal dysfunction, malignancies and organ transplants are positive predictors of the presence of iMLSB among CA-MRSA [65], however, we did not assess those comorbidities in our study participants. These inducible clindamycin-resistant strains have hindered the effective use of clindamycin [66–68]. This spotlights the importance of a simple D-zone test if erythromycin-resistant and clindamycin-sensitive isolates are isolated. Furthermore, if clindamycin is used for treatment against iMLSB, regular follow-up and monitoring for probable treatment failure or relapse are needed [69].

All the phenotypic MRSA isolates in this study were *mecA* gene positive and most of the MRSA strains harbored the *PVL* gene. We observed that all the MRSA strains from PLHIV but not all MRSA from people were *PVL* gene positive. This could be because a higher proportion of chronic skin disorders or chronic wounds are seen among the HIV population in contrast to HIV-negative people, a higher occurrence of chronic skin disorders has been linked to higher CA-MRSA colonization among PLHIV [70,71]. Despite the phenomenon of a combination of *PVL* and methicillin resistance in *S. aureus* being a comparatively recent process, it has been emerging rapidly and has already widely spread among *S. aureus* clones [20,72,73]. Because the consequences of infection with *PVL*-positive strains tend to be more severe than those of *PVL* negative strains [74], propagation of clones combining methicillin resistance and *PVL*-positive strains needs to be properly managed. The lower resistance of MRSA to cotrimoxazole and clindamycin suggests the colonization by CA-MRSA [17,43,58]. Although the reason is not established, previously published literature shows an increasing trend of community-associated MRSA among PLHIV [13,37,38,46].

## Conclusion

No significant difference in MRSA nasal colonization in PLHIV and healthy controls was found in this study region. As a longer stay in the hospital could increase the risk of MRSA colonization, PLHIV should be managed in a way that reduces the duration of stay in the hospital. A well-monitored ART should be encouraged and continued for PLHIV. An effective antibiotic regulatory program should be implemented to prevent the spread of MRSA and MDR-*Staphylococcus aureus* among PLHIV and even among seemingly healthy individuals.

**Summary points**

- Although *Staphylococcus aureus* is a common colonizer of the anterior nares, it can cause life-threatening infections among immunocompromised individuals, such as people living with HIV (PLHIV).
- PLHIV are at a greater likelihood of methicillin-resistant *S. aureus* (MRSA) infection than the general population, thus this study was intended to compare the incidence of MRSA in PLHIV and apparently healthy people from the Kathmandu Valley, Nepal.
- Out of 400 nasal swabs analyzed, 42 *S. aureus* were isolated and among all isolates, 17 were from PLHIV and 25 were from people from the Kathmandu Valley.
- Among 200 PLHIV enrolled, most of PLHIV were under antiretroviral therapy (ART) for 6 months or more, 16% of PLHIV had a recent history of hospitalization and 9% of them had a CD4 cell count of less than 200 cells/μl.
- Out of 42 *S. aureus* isolates, 27 isolates were MDR, of which 17 isolates were MRSA. There was no statistically significant difference in the prevalence of MRSA and MDR among the study group.
- The D-test showed that nine of total *S. aureus* isolates were inducible macrolide-lincosamide streptogramin B (iMLSB) including three from PLHIV and six from healthy controls.
- All the phenotypic MRSA isolates from both the study groups harbored the *mecA* gene, and the *PVL* gene was detected in 21 isolates. There was no statistically significant difference in the presence of *PVL*-positive MRSA between the two groups.
- The MRSA nasal colonization was found to be the highest in the participants with an ART duration of less than 6 months. The longer ART duration significantly lowers the odds of nasal colonization by MRSA.
- The MRSA nasal colonization rate was statistically significant in both cases of PLHIV and healthy controls with a history of hospitalization within the past 6 months.
